# The activity of hydrolytic enzymes and antibiotics against biofilms of bacteria isolated from industrial-scale cooling towers

**DOI:** 10.1186/s12934-024-02502-1

**Published:** 2024-10-16

**Authors:** Marcus Vinícius Dias-Souza, Andrea Lima Alves, Sérgio Pagnin, Andrea Azevedo Veiga, Ihtisham Ul Haq, Wadi B. Alonazi, Vera Lúcia dos Santos

**Affiliations:** 1 Applied Microbiology Laboratory, Microbiology Department, Instituto de Ciências Biológicas, Universidade Federal de Minas Gerais, Avenida Antônio Carlos 6627, C.P. 486, Belo Horizonte, Minas Gerais 31270-901 Brazil; 2 Research and Development Center (CENPES), Petróleo Brasileiro S.A., Rio de Janeiro, Brazil; 3 Department of Physical Chemistry and Technology of Polymers, Silesian University of Technology, M. Strzody 9, Gliwice, 44-100 Poland; 4 Joint Doctoral School, Silesian University of Technology, Akademicka 2A, Gliwice, 44-100 Poland; 5 Programa de Pós-graduação em Inovação Tecnológica, Universidade Federal de Minas Gerais, Belo Horizonte, Minas Gerais 31270-901 Brazil; 6 Health Administration Department, College of Business Administration, King Saud University, Riyadh, Saudi Arabia

**Keywords:** Cooling tower, Industries, Bacteria, Enzymes, Antibiotics, Biofilms

## Abstract

**Background:**

Cooling towers (CTs) are crucial to myriad industrial processes, supporting thermal exchange between fluids in heat exchangers using water from lakes and rivers as coolant. However, CT water can sometimes introduce microbial contaminants that adhere to and colonize various surfaces within the CT system. These microorganisms can form biofilms, significantly hindering the system’s thermal exchange efficiency. Current treatment strategies employ oxidizing biocides to prevent microbial growth. However, despite their affordability, they do not eliminate biofilms effectively and can lead to corrosive damage within the system. Herein, we aim to devise an anti-biofilm strategy utilizing hydrolytic enzymes (such as α-amylase, glucoamylase, pectin-lyase, cellulase, protease, and DNase) alongside antibiotics (including meropenem, ciprofloxacin, gentamicin, erythromycin, chloramphenicol, and ceftriaxone) to combat microbial growth and biofilm formation in cooling systems.

**Results:**

All enzymes reduced the development of the biofilms significantly compared to controls (*p* < 0.05). The polysaccharidases exhibited biomass reduction of 90%, except for pectin-lyase (80%), followed by DNAse and protease at 43% and 49%, respectively. The antibiotics reduced the biofilms of 70% of isolates in concentration of > 2 mg/mL. The minimal biofilm eradication concentration (MBEC) lower than 1 mg/mL was detected for some 7-day-old sessile isolates. The enzymes and antibiotics were also used in combination against biofilms using the modified Chequerboard method. We found six synergistic combinations, with Fractional inhibitory concentrations (FIC) < 0.5, out of the ten tested. In the presence of the enzymatic mixture, MBECs presented a significant decrease (*p <* 0.05), at least 4-fold for antibiotics and 32-fold for enzymes. Moreover, we characterized high molecular weight (> 12 kDa) exopolysaccharides (EPS) from biofilms of ten isolates, and glycosyl composition analysis indicated a high frequency of glucose, mannose, erythrose, arabinose, and idose across isolates EPS contrasting with rhamnose, allose, and those carbohydrates, which were detected in only one isolate.

**Conclusion:**

The synergistic approach of combining enzymes with antibiotics emerges as a highly effective and innovative strategy for anti-biofilm intervention, highlighting its potential to enhance biofilm management practices.

**Supplementary Information:**

The online version contains supplementary material available at 10.1186/s12934-024-02502-1.

## Introduction

Cooling towers (CTs) serve as crucial heat exchange systems widely utilized in various sectors, including oil refineries, hospitals, power generation facilities, and the food and pharmaceutical industries, facilitating the removal of excess heat from processes or equipment [1]. CTs discharge heat from systems by utilizing water to maintain optimal internal temperatures. CTs typically use low-cost water sources such as lakes or rivers or reused water [2]. However, even when treated, these water sources can bring high levels of organic compounds and microorganisms to industrial systems [3–5]. These compounds, along with the constituents of additives such as anti-corrosives and anti-scaling agents added to the tower waters during operation, or those coming from the atmosphere in the case of open towers can constitute nutrient sources for microbial growth. These conditions create an ideal environment for establishing microorganisms in planktonic or sessile forms organized in biofilms [6–8]. Microbial biofilms are complex structures of microorganisms embedded in a self-produced matrix consisting of extracellular polymeric substances, mainly polysaccharides, proteins, lipids, and extracellular DNA [6]. They protect microorganisms from environmental factors such as low pH, high temperature, and antimicrobials [7, 8] Microbial biofilms on CT surfaces can lead to corrosion, thereby diminishing the CTs efficiency [5, 9, 10] Additionally, the water flow in CTs aids in spreading bacteria throughout the surrounding area, increasing the risk of microbial contamination [11, 12].

Current microbial growth control methods in CTs utilize biocides, including chlorine and bromine-based products, which pose risks to human health and the environment and have frequently been reported as ineffective in controlling microbial [13–15] Due to their limited biodegradability, chlorine, and bromine-based products’ environmental persistence and bioaccumulation can present significant risks to wildlife and plant life [16]. Such scenarios require promising antimicrobial strategies to avoid biofilm formation [17, 18]. Given the harmful effects of current antimicrobial methods, enzymes with antimicrobial properties are recognized as effective and environmentally friendly alternatives for biofilm prevention. This shift highlights the need for safer biocontrol strategies in microbial management [19]. Nature has diverse sources of antimicrobial enzymes that help organisms avoid random microorganisms; however, currently, antimicrobial strategies employ these enzymes against microbial systems. Enzyme-based therapies exhibited promising antimicrobial results in industrial systems, cleaning agents, disinfectants, and detergents [11, 20]. The most frequently reported enzymes with antimicrobial potential are proteases that hydrolyze bacterial protein enzymes [21], lysozymes [22], alginate, and amylases that degrade polysaccharides [23]. The other promising antimicrobial enzymes are subtilisins [24] and bacteriophage-derived enzymes [25].

Antimicrobial enzymes are highly advantageous due to their catalytic efficiency and specific action across diverse temperature ranges. These characteristics render them highly effective for various industrial uses as powerful antimicrobial agents [26]. Enzymes exhibit diverse mechanisms for inhibiting bacterial growth and disrupting biofilms in several ways; (1) hydrolysis of adhesion proteins, which are necessary for bacterial attachment [27], (2) permeabilization of the bacterial membrane [28], (3) hydrolyzing of bacterial polysaccharides [19], (4) production of superoxide anions [29], (5) inhibition of the quorum sensing [30] and (6) rupture of matrix of mature biofilms resulting in dispersion them besides increasing sensitivity of the biofilm to antimicrobial agents [31].

This is the first study that uses a combined approach of enzyme and antimicrobials-based therapies for removing bacterial biofilms in industrial-scale CTs, which have yet to be extensively explored. In this pioneering study, we aimed to evaluate the efficacy of various enzymes, both alone and in combination with antimicrobial drugs, in controlling bacterial biofilms within the context of a Brazilian oil refinery plant’s CTs system. Additionally, we conducted a comprehensive characterization of the high molecular weight EPS extracted from these biofilms, analyzing their composition and linkage patterns.

## Materials and methods

### Sampling and bacterial isolation

Based on their demonstrated biofilm-forming capabilities, we selected a cohort of 50 bacterial isolates of 18 planktonic and 32 sessile bacteria. These isolates were chosen from a larger pool of 160 collected from the water samples of two industrial-scale CTs (CTI and CTII) at an oil refinery in Brazil. CTI receives its water supply from a secondary effluent generated within the Industrial Waste Treatment Plant of the oil refinery. This water undergoes a rigorous treatment process in a model reuse unit, beginning with chlorination using sodium hypochlorite and filtration through sand and galvanized carbon filters. Subsequently, the water undergoes treatment via reverse electrodialysis.

In contrast, the water-feeding CTII originates from an artificial lagoon and goes through a distinct treatment regimen. Initially, it undergoes clarification and chlorination, with pre-chlorination optimizing coagulation and clarification processes. Following clarification, an additional chlorine dosage is administered to maintain the free residual chlorine content within the 0.5 to 1.0 mg/L range. Subsequently, the water is directed through sand filters to eliminate particulate matter before being supplied to the tower.

For planktonic bacteria isolation, sterile amber bottles containing 0.1% sodium thiosulfate, which neutralizes the action of residual chlorine, were used to collect 1 L of water from the basin of the towers. Sessile bacteria were obtained from glass slides (76 mm x 26 mm x 1.3 mm) immersed in the water basins with the help of plastic holders. After 7, 14, and 21 days, slides were retrieved from the basin of CTs and in sterile distilled water to remove non-adhered microorganisms. The bacteria adhered to surfaces were detached using sonication (3 cycles of 2 minutes, 40 kHz) and vortexing (60 s) at maximum speed. In both isolation strategies, serial dilutions ranging from 10^− 1^ to 10^− 7^ were performed and plated on Plate Count agar (Oxoid) for counting and isolation. After incubation at 37ºC for 24–48 h, all different morphotypes were characterized and counted (CFU/mL or CFU/cm^2^). Different morphotypes obtained were identified by sequencing the 16 S region of the rRNA gene. Those used in this study belonging to genera *Bacillus* (30 isolates) (*Bacillus circulans*,* Bacillus* sp *Subtilis* Group; *Bacillus* sp *Cereus* Group), *Enterobacter* (2 isolates) *(E. hormaechei)*,* Brevibacterium (B. halotolerans)*,* Kluyvera* (1 isolate) *(K. cryocrescens)*,* Acinetobacter* (9 isolates) *(A. junii*,* A. radioresistens*, and *A. haemolyticu)*,* Stenotrophomonas* (1 isolate) *(S. maltophilia)*,* Pseudomonas* (1 isolate) *(P. stutzeri)*,* Staphylococcus* (1 isolate) *(S. epidermidis)*, *Lysinibacillus (L. sphaericus)*,* Exiguobacterium* (1 isolate) *(E. mexicanum)*,* Geobacillus* (1 isolate) *(G. stearothermophilus*). The full relation of species can be found in supplementary tables.

### Antibiofilm activities of enzymes in vitro

#### Biofilm formation

Overnight cultures of the isolates in nutrient broth (Difco) were centrifuged, washed, and adjusted to reach 0.5 McFarland scale turbidity in nutrient broth. Aliquots (200 µL) of the suspensions were dispensed in untreated sterile 96-well flat-bottom polystyrene plates (Sarstedt, Germany), using fresh broth as a negative control. After incubation at 35 ± 2 °C overnight, the wells were gently aspirated and washed three times with sterile saline solution (0.85% NaCl).

#### Biofilm viability and rupture assays

Different enzymes such as α-amylase (AMG, 480 U/mL), glucoamylase (BAN, 300 U/mL), and pectin-lyase (Ultrazym, 3000 U/mL) were kindly provided by Novozyme (Bagsvaerd, Denmark). Cellulase (0.8 U/mg), protease (2.4 U/mg), and DNAse (2000 U/mg) were purchased from Sigma. All these enzymes’ antimicrobial activity was tested against the isolated bacteria. To assess the antimicrobial assay, enzyme solutions were diluted in sterile PBS (NaCl 8,0 g/L, KCl 0,2 g/L, Na_2_HPO_4_ 1,15 g/L, KH_2_PO_4_ 0.2 g/L, pH 7) to reach the concentration of 100 U/mL and added to wells with overnight formed biofilms. The chosen pH value was based on the pH levels observed in the water of the CT basins, where the enzymes are intended to control biofilms. The enzyme concentration was selected based on prior experiments (data not shown), in which concentrations ranging from 5 to 100 U/mL (increasing by two factors) and exposure times of 4, 8, 12, and 18 h were tested. After dispensing the enzymes in the wells, the plates were incubated at 37 ºC, and following, the wells were gently aspirated and washed three times with 100 µL of sterile PBS.

Biofilm viability was assessed using resazurin as a metabolic activity indicator. Fluorescence (λ_ex_570 nm and λ_em_590 nm) of resorufin was measured by a microplate reader (Varioskan, Thermo Fisher) using a sterile medium and resazurin as a control. Reads were taken in triplicate for each strain and registered as arbitrary fluorescence units (AFU) [32]. The phenol-sulfuric acid method [33] was used to estimate the remaining total carbohydrates to assume biofilm inhibition after the treatment with polyssacharidases. Glucose was standard, and photometric reads were made at 490 nm in the microplate reader. After DNAse and protease treatments, biofilm rupture was inferred with crystal violet staining [33]. The wells were washed with sterile saline and exposed to 200 µL of methanol to fix the biofilms for 20 min. Methanol was then gently removed, and the wells were exposed to 150 µL of a 1% crystal violet solution for 15 min. Next, the dye was removed, and the wells were washed twice. The wells were then exposed to 150 µL of 96 °GL ethanol for 15 min. Treated and non-treated plates were analyzed in a microplate reader using photometric reads at 600 nm [32]. Absorbance values in both tests are expressed as the average of at least three replicates ± standard deviation.

### Antibiofilm activities of antibiotics

This assay was performed in triplicate using untreated 96-well polystyrene plates as described elsewhere [32]. Overnight cultures of the isolates were used for biofilm preparation, as described in the previous section. The antibiotics such as meropenem, ciprofloxacin, gentamicin, erythromycin, chloramphenicol, and ceftriaxone (Sigma) were tested against bacterial biofilms. All antibiotics were prepared as stock solutions in dimethyl sulfoxide (DMSO) (Sigma) to a concentration of 40 mg/mL. Then, they were serially diluted in PBS to reach concentrations ranging from 2 mg/mL to 15.62 µg/mL. Before the antibiofilm assay, the biofilms were washed, and aliquots of 200 µL of the diluted drugs were transferred to the wells in triplicate. Plates were then incubated at 37 °C overnight. After incubation, the medium aspired, the plates were washed three times with sterile saline solution (0.85% NaCl), and resazurin was added as described. The minimal biofilm eradication concentration (MBEC) was considered the lowest concentration in which resazurin was not converted to resorufin.

### Enzymes and antimicrobial drugs as combination therapy

Enzymes and antibiotics were combined to use strong antimicrobial therapy against the biofilms using the modified approach of the Chequerboard method [34]. The following combinations of antimicrobials and enzymes were chosen considering the best results of the previous experiments against the isolates: gentamicin-AMG (*Elizabethkingia meningosptica* isolates), chloramphenicol-AMG (*Acinetobacter junii* isolates), Meropenem-AMG (*Klyuvera cryocrescens* isolates), ciprofloxacin-BAN (*Exiguobacterium mexicanum* isolates), cephalexin-BAN (*Stenotrophomonas maltophilia* isolates), Ceftriaxone-AMG (*Pseudomonas stutzeri* isolates), chloramphenicol-Celullase (*Lysinibacillus sphaericus* isolates), cephalexin-AMG (*Acinetobacter haemolyticus* isolates), meropenem-BAN (*Enterobacter hormaechei* isolates), erythromycin-cellulase (*Bacillus sp.* isolates).

Solutions of the antibiotics were serially diluted in PBS with concentrations ranging from 4 mg/mL to 0,0625 mg/mL in the vertical direction. Enzyme solutions were prepared in the PBS buffer, with concentrations ranging from 400 to 0.78 U/mL (considering the activities described in Table) in the horizontal direction. Each well with formed biofilm was filled with 100 µL of each concentration of drugs and enzymes, except wells with biofilms exposed only to drugs at different concentrations under test or only to enzymes to varying concentrations under test. The plates were incubated for 18 h at 37 ºC; then resazurin was added as already described. Fractional inhibitory concentrations (FICs) were then calculated, and the FIC index was obtained as previously described [34]. Values were interpreted as synergistic if FIC ≤ 0.5, additive if 0.5 < FIC < 1.0, indifferent if 1 < FIC < 2, and antagonistic if FIC > 2.0. Isobolograms were plotted to confirm the FIC results.

### Biofilm EPS characterization

#### Biofilm EPS extraction

Biofilms grown for EPS purification were prepared by inoculating 10 ml of bacterial suspension with cell density corresponding to 0.5 McFarland scale produced in nutrient broth media per each well of untreated sterile 96-well flat-bottom polystyrene plates (Sarsted, Germany). Biofilms were grown at 35 ± 2 °C with shaking for three days, followed by incubation without shaking for four days to let the formation of mature biofilms [35]. These incubation conditions were used to obtain biofilms on the bottom and the wall of wells, maximizing the production of EPS. Then, the media were gently aspirated, and the mature biofilms were washed three times with sterile saline solution (0.85% NaCl) and transferred to 15 mL tubes. After centrifugation at 10,000×g for 10 min at four °C, the supernatant was discarded, and pellets were suspended in saline solution. For EPS extraction, 6 µl of formaldehyde (36.5% solution) was added to each 1 mL of sludge to fix the cells and prevent cell lysis during subsequent steps. The mixture was incubated at room temperature with gentle shaking (100 rpm) for 1 h. Aliquots 400 uL of 1 M NaOH were added for each 1 ml of biofilm suspension and incubated at room temperature, with 200 rpm shaking, for 3 h to extract EPS. Cell suspensions were then centrifuged (16,800×g) for 1 h at 4 °C. The supernatant containing soluble EPS was filtered through a 0.2 μm filter and dialyzed against distilled water using a 10–12 kDa molecular weight cut-off (MWCO) membrane for 18 h at room temperature.

#### Partial purification of exopolysaccharides

Trichloroacetic acid (TCA) was added (20% w/v, 4 °C) to extracted EPS solutions in the same volume to precipitate proteins and nucleic acids. After 30 min, the solution was centrifuged (16,800×g) for 1 h at four °C, the supernatant was collected, 1 volume of 95% ethanol was added, and the mixture was placed at − 20 °C for 18 h to precipitate EPS away from lipids. The tubes were then centrifuged (16,800×g) for 1 h at 4 °C, and the exopolysaccharide pellet was resuspended in Milli-Q water, lyophilized, and weighed. Fractions of lyophiles (1 mg/mL) were tested for the presence of carbohydrates by the phenol-sulfuric acid using glucose according to the protocol of [33] and lipids using the method of Bligh and Dyer [36]. The Bradford method [37] was used to analyze total proteins, adapted to be carried out in 96-well microplates, using bovine serum albumin as standard. Analyses were conducted using a Nanodrop spectrophotometer at 260 nm (ThermoFisher) to determine total nucleic acids.

#### Monosaccharide composition analysis

EPS derivatization followed the protocol described elsewhere [36]. Twenty mg of EPS were hydrolyzed with trifluoroacetic acid 2 mol/L (2 ml) at 120 °C for two h. Hydrolysis is followed by removing TFA using six washes with methanol (PA) and drying in an N2 atmosphere. Each tube was added 100 µL of sodium borohydride (NaBH4) and remained closed and at room temperature for 30 min. To remove NaBH_4_, a glacial acetic acid solution (5% v/v methanol) was dripped into each bottle, and the contents were then dried in an N2 atmosphere. This step was repeated thrice until no more effervescence was noticed upon dripping. Then, 200 µL of a sodium acetate solution (PA, 1 mg/mL) was dispensed in acetic anhydride (PA). The tubes were heated at 100 °C for 20 min and dried by heating at 100 °C and with an N_2_ atmosphere. Three washes were carried out with xylene to remove acetic anhydride and dried in an N_2_ atmosphere. Ten washes were carried out with ethyl acetate to remove xylene and dried in an N_2_ atmosphere. To the final content, with the appearance of an amorphous mass and in shades of grey, 1 mL of ethyl acetate was added and homogenized. Then, 1 mL of Milli-Q water was added and homogenized again to partition between the aqueous and organic phases containing the alditols. After phase separation, the upper portion (organic phase) was collected, transferred to new bottles, and subjected to drying with N_2_. With a caramelized appearance, the final product was analyzed by GC-MS (QP2010-SE, Shimadzu) equipped with a column (PTE-5, Sigma, 0, 25 μm, 30 m x 0,32 mm). The carrier gas used was helium, and the temperature of the injector and detector were kept at 100 and 250 °C, respectively. The temperatures of the GC interface with the EM and the ion source were set to 230 °C. The column operated at a flow rate of 1.17 mL/min, at a pressure of 13.7 kPa, and a linear speed of 40.2 cm/s. Compounds detected in the 35 to 700 Da range were identified by comparison with the system database (NIST/EPA/NIH Mass Spectral Library, National Institute of Standards and Technology, USA). Monosaccharides were identified by comparing them with the monosaccharides of Sigma.

### Statistical analysis

Microsoft Excel calculated the mean and standard deviation values, and the Levene test evaluated heteroscedasticity. Data normality was assessed by the Shapiro-Wilk test. Differences between the means of enzyme activity on biofilms were assessed by the Kruskal-Wallis test, followed by Dunn’s test. A level of *p* < 0.05 was accepted as significant, and *p* < 0.01 was accepted as highly effective. Data were analyzed in the statistical package Minitab 17 for Windows.

## Results

### Enzymes effects on the bacterial biofilms

**Fig. 1:**
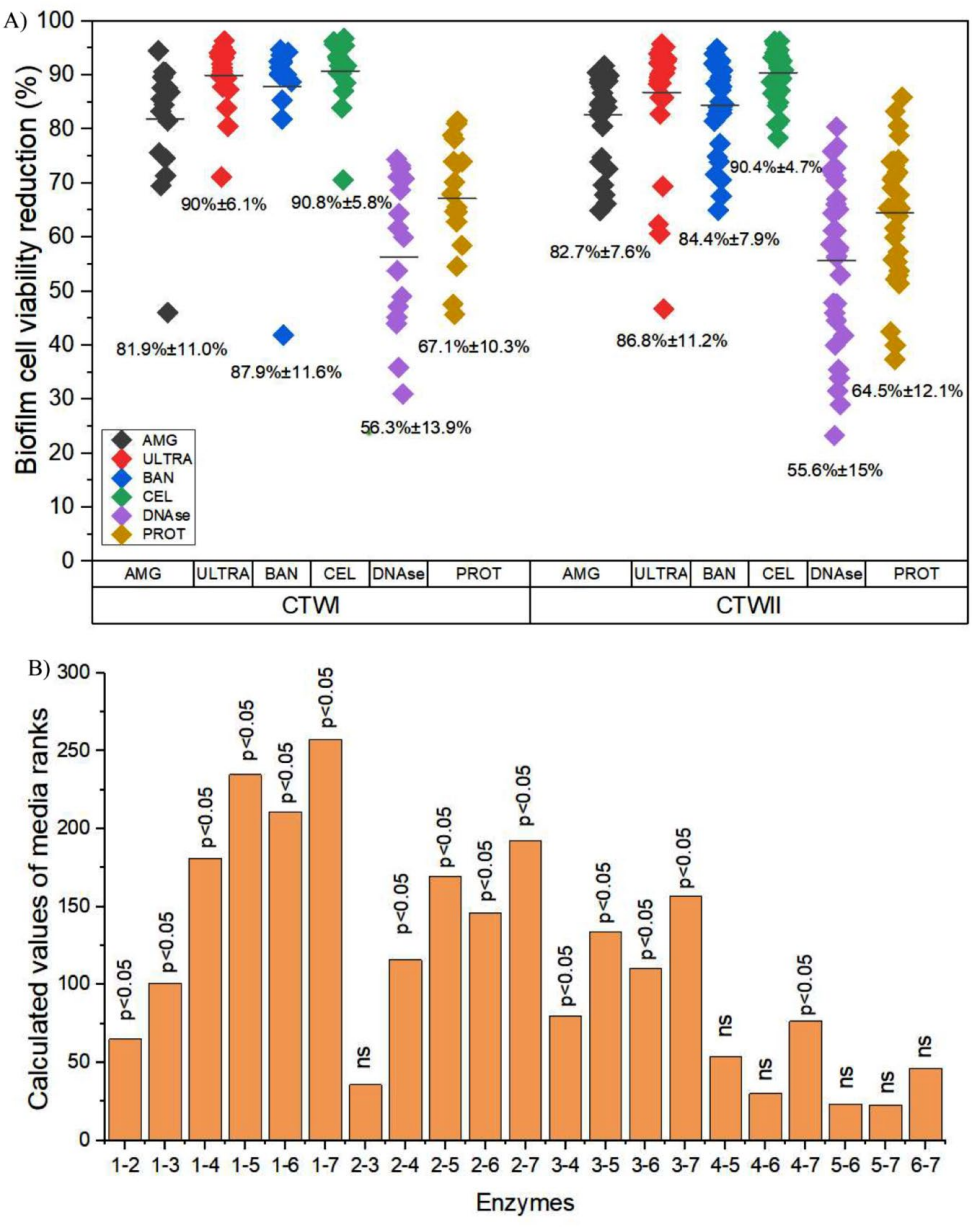
Effects of enzymes on biofilms cell viability reduction (**A**). Viability was expressed as fluorescence arbitrary units (FAU) measured at least three independent experiments. The points represent mean values for each bacterium evaluated, and the numbers indicate the mean followed by the standard deviation for all isolates from each enzyme for CT. (**B**) Comparison of the reduction in the cell viability of biofilms treated with enzymes using the Kruskal Wallis method. 1 - Control, 2 - DNAse, 3 - Protease, 4 - AMG, 5 - Ultra, 6 - BAN, 7 - Cellulase. NS: not significant

 All enzymes reduced the viability of the biofilms significantly compared to controls (*p* < 0.05). However, the average percentage of biofilm viability reduction by polysaccharidases was higher than that observed after DNAse and protease treatments (Fig. 1A; Table 1). Cellulase presented the best results (*p* < 0.05) (Fig. 1B). The variance analysis indicated no effect of biofilm age present in glass slides used in the bacterial isolation (7, 14, or 21 days), source (CT), or isolation pattern (sessile or planktonic) on the activity of the enzymes.

**Table 1:** Effects of enzymes on biofilms viability. Fluorescence data (λex: 570 nm; λem: 590 nm) is presented as averages of at least three independent experiments. Data in enzymes and control columns is expressed as fluorescence arbitrary units (FAU). The SD values are less than 13% of the media

Bacterial origin		Identity	Control	DNAse	Protease	AMG	Ultra	BAN	Cellulase
**Source (CT)**	**Growth profile**		FAU	FAU	FAU	FAU	FAU	FAU	FAU
CT I	Planktonic (14 days)	*Bacillus sp.* Grupo Cereus 26	2286.66	610.04	594.83	213.13	182.20	176.85	87.01
	Palnktonic (21 days)	*Kluyvera cryocrescens* 40	1623.71	857.12	601.50	233.79	95.13	140.32	184.59
		*Lysinibacillus sphaericus* 116	1529.97	837.94	800.19	188.90	80.59	113.72	449.90
	Sessile (7 days)	*Bacillus sp.* Grupo Cereus 23	1720.12	658.33	512.10	317.74	116.47	178.07	78.55
		*Acinetobacter radioresistens* 104	1607.42	819.74	338.00	391.15	258.15	164.98	258.24
		*Acinetobacter beijerinckii* 107	1509.63	845.06	398.60	184.26	190.94	94.14	124.32
		*Bacillus sp.* Grupo Subtilis 131	1312.54	904.88	594.83	170.66	48.42	192.45	108.18
		*Bacillus sp.* Grupo Cereus 138	1927.48	769.56	677.81	238.07	93.66	141.33	184.60
		*Bacillus sp.* 111	1350.45	864.87	476.36	386.23	125.94	245.00	131.44
	Sessile (14days)	*Bacillus sp.* Grupo Cereus 4	2145.13	625.52	395.00	652.74	417.75	214.42	189.89
		*Bacillus sp.* Grupo Cereus 28	2823.18	811.84	613.84	152.80	158.66	273.89	225.55
		*Acinetobacter junii* 41	1775.80	820.93	610.05	184.59	187.60	94.18	126.01
		*Bacillus sp.* Grupo Subtilis 55	1427.66	659.02	371.20	238.07	93.66	141.33	184.60
		*Stenotrophomonas maltophilia* 94	2285.91	811.90	946.60	286.53	279.93	172.41	167.25
		*Bacillus sp* Grupo Cereus 95	2204.67	688.36	777.20	340.52	190.20	127.59	94.66
		*Acinetobacter junii* 97	1267.74	811.70	687.70	683.52	365.80	737.41	82.85
	Sessile (21 days)	*Bacillus sp.* Grupo Subtilis 17	2523.71	646.47	806.08	238.80	147.57	247.61	169.13
		*Bacillus sp.* 43	2420.79	659.79	459.29	613.02	245.47	272.39	77.71
CT II	Planktonic (7 days)	*Bacillus sp.* 30	1520.36	638.90	677.81	145.73	146.76	381.85	290.77
		*Bacillus sp* Grupo Cereus 66	1836.05	798.34	474.76	184.26	190.94	94.47	124.32
		*Staphylococcus epidermidis* 72	1611.22	893.36	925.50	419.52	188.86	127.52	84.76
		*Enterobacter hormaechei* 108	1483.83	774.82	385.53	372.97	137.60	134.90	233.22
		*Acinetobacter haemolyticus* 125	1278.11	843.86	545.59	189.07	76.90	217.25	172.75
		*Bacillus sp.* Grupo Subtilis 135	2100.00	435.00	400.10	207.00	300.00	130.10	110.21
	Planktonic (14days)	*Elizabethkingia meningoseptica* 8	2830.22	769.69	398.60	232.16	136.71	244.68	167.96
		*Acinetobacter haemolyticus* 9	1369.14	796.58	385.53	375.10	94.07	124.79	102.87
		*Bacillus sp* Grupo Subtilis 10	2386.47	658.93	805.00	207.41	184.86	182.28	186.08
		*Acinetobacter junii* 20	1814.46	748.23	800.19	218.50	256.42	270.60	157.29
		*Bacillus sp.* Grupo Cereus 44	1840.58	442.10	567.72	320.91	262.97	213.12	98.86
		*Geobacillus stearothermophilus* 45	2144.51	725.32	415.32	326.50	655.24	630.73	241.85
		*Bacillus sp* Grupo Subtilis 49	2011.59	879.27	697.42	309.16	756.61	455.84	213.96
		*Enterobacter hormaechei* 152	2117.90	696.72	610.05	243.37	140.04	128.72	135.66
	Planktonic (21 days)	*Bacillus sp.* Grupo Cereus 157	1865.72	825.957	459.290	275.992	408.149	432.419	152.376
	Sessile (7 days)	*Bacillus sp.* 25	1578.91	612.89	545.59	257.39	116.34	177.69	80.89
		*Bacillus sp.* Grupo Cereus 38	1464.30	763.88	675.36	165.37	61.76	226.08	108.22
		*Exiguobacterium mexicanum* 63	2125.46	891.97	611.72	683.52	365.85	743.77	79.99
		*Bacillus sp* Grupo Cereus 86	2419.27	560.99	966.28	406.09	167.48	176.89	91.05
		*Bacillus circulans* 102	1997.24	695.49	423.22	309.87	175.20	327.92	268.70
		*Bacillus sp.* 106	1227.65	840.10	395.00	238.07	93.66	141.33	184.60
		*Pseudomonas stutzeri* 110	1712.23	924.57	805.59	330.59	673.81	208.15	141.22
	Sessile (14 days)	*Bacillus sp.* 1	2026.13	578.13	338.00	243.88	224.11	222.48	112.74
		*Bacillus sp* Grupo Cereus 11	1853.90	870.84	476.36	204.59	184.72	343.42	137.03
		*Acinetobacter junii* 34	1260.30	755.40	601.61	189.08	76.90	217.26	173.22
		*Bacillus sp.* 83	1785.57	755.99	644.20	202.89	184.66	464.91	128.36
		*Bacillus sp.* 112	1344.07	866.91	806.08	144.06	130.07	381.80	290.74
		*Acinetobacter haemolyticus* 118	1339.40	1027.18	512.10	469.64	154.34	137.61	247.93
		*Bacillus sp.* Grupo Subtilis 140	2256.44	664.05	601.61	683.52	320.80	731.07	95.85
		*Bacillus sp.* 143	2428.46	863.36	675.36	386.37	127.42	254.68	141.70
		*Bacillus sp.* Grupo Subtilis 147	2055.75	401.51	601.50	303.87	175.20	327.92	265.36
	Sessile (21 days)	*Bacillus sp.* 91	1815.30	703.04	1134.71	239.67	136.77	230.99	164.33

**Fig. 2:**
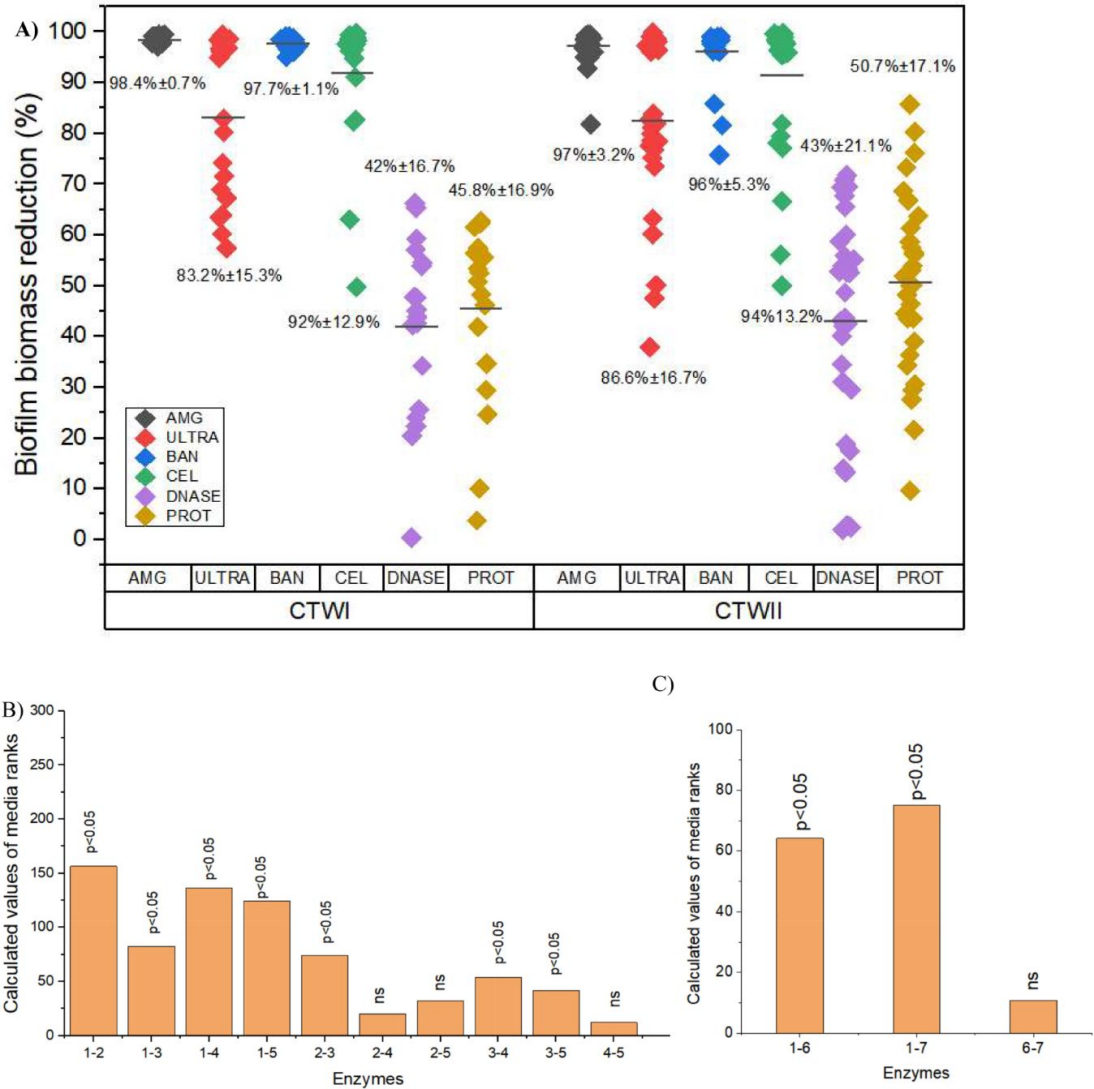
Biomass reduction after treatment of biofilms with enzymes. Polysaccharidases activity was assessed through phenol-sulphuric method and DNAse and Protease by Crystal violet staining. The points represent mean values for each bacterium evaluated, and the numbers indicate the mean followed by the standard deviation for all isolates from each enzyme for CT. Comparison of the reduction values in biomass of biofilms treated with polysaccharidases (**B**) or DNAse and protease (**C**) using the Kruskal Wallis method. 1 - Control, 2 - AMG, 3- Ultra, 4 - BAN, 5 – Cellulase, 6- DNAse, 7- Protease NS: not significant

Regarding biofilm biomass, all polysaccharidases were able to reduce the total carbohydrate content significantly compared to controls (*p* < 0.05) (Fig. 2A and B; Table S1). The average percentage of biomass reduction was superior to 90%, except for pectin-lyase (Ultrazym), for which the average biomass reduction was around 80%. α-amylase (AMG) presented a higher efficiency for biofilm disruption (*p* < 0.05). There was a significant difference in enzyme activity against biofilms formed by planktonic or sessile bacteria collected after seven days in CT (*p* < 0.05). However, this difference was not observed for planktonic and sessile cells in 14 and 21 days biofilms. Also, there was no difference in enzyme activity concerning the genera identified in this study, except for the *Bacillus* strains, to which AMG presented higher efficacy (*p* < 0.05). Similarly, DNase and protease were influential in the biofilm removal when compared to the control group (*p* < 0.05), albeit there was no significant difference between the results of these two enzymes (Fig. 2C). The rupture effectiveness of these enzymes was lower than that observed for polysaccharidases. The average percentages of biomass reduction for DNase were 42% ± 16.7% for bacterial isolates from CTI and 43% ± 21.1% for isolates from CTII. For protease, the average percentages were 45.8%± 16.9% and 50.7 ± 17.1% for CTI and CTII, respectively (Fig. 2A). There was no significant difference in the activity of these enzymes in disruption of biofilms regarding the age of biofilms present on the slides from which the bacteria were isolated, source (CT), genera, or their isolation pattern (sessile or planktonic). Biofilm biomass indirect measurements expressed as glucose concentration on the polysaccharidases tests and absorbance reads at 590 nm for DNAse and protease tests can be seen in Tables S1 and S2.

### Susceptibility of bacterial biofilms to antibiotics

**Fig. 3:**
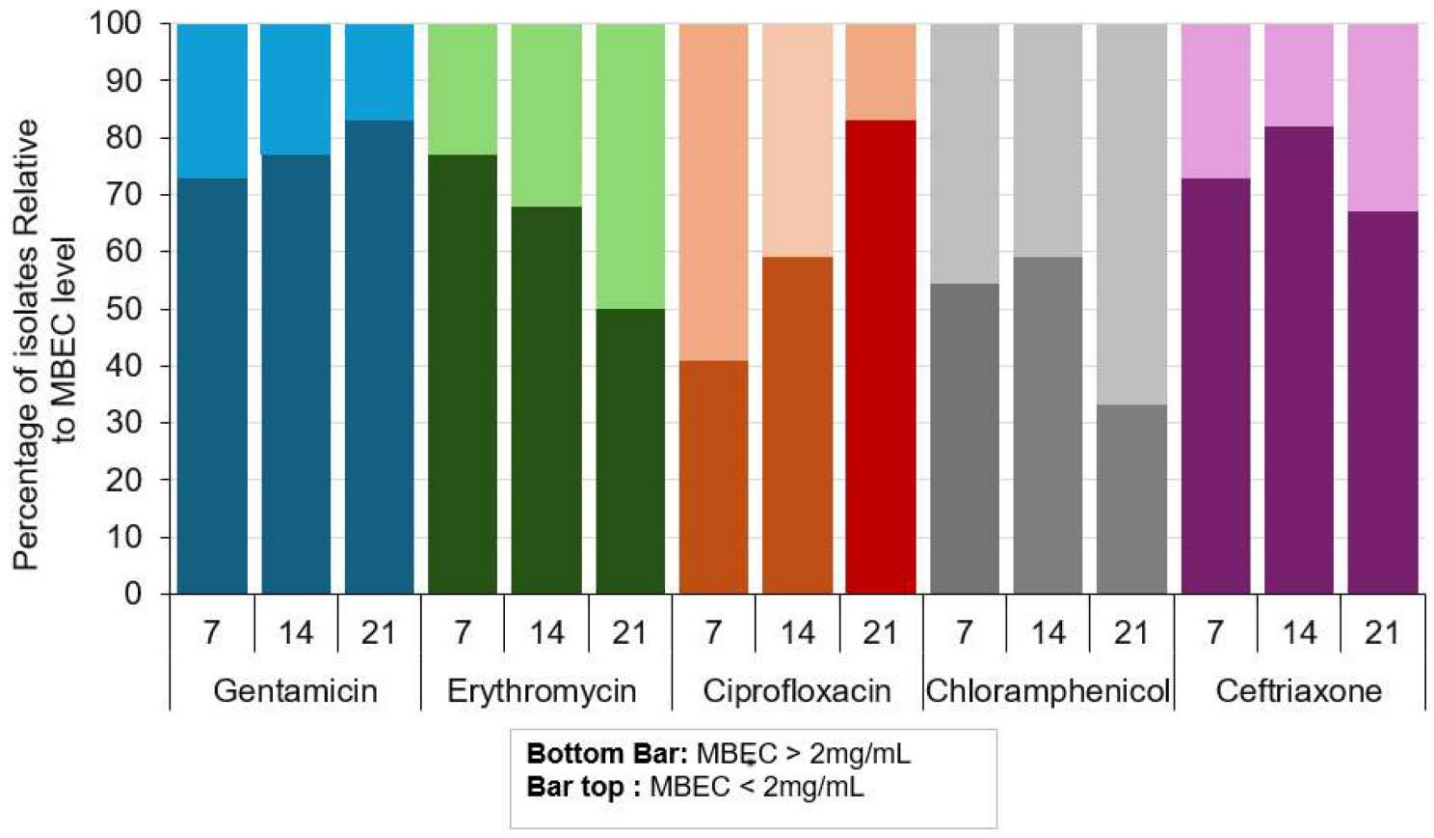
Percentual of bacteria with MBEC > 2 mg/mL and MBEC < 2 mg/mL considering biofilm age present on glass slides used for bacterial isolation

The biofilms of approximately 70% of isolates exhibited susceptibility to antibiotics in the concentration of > 2 mg/mL for gentamicin, erythromycin, and ceftriaxone. Biofilm susceptibility of nearly 60% of isolates was > 2 mg/mL for ciprofloxacin and chloramphenicol (Fig. 3; Table S3). For all isolates (100%), the biofilm susceptibility to cephalexin and meropenem was more significant than 2 mg/mL. MBEC lower than 1 mg/mL was detected for two 7-days-old sessile isolates from tower CTI: *Pseudomonas stutzeri* 110 (Ciprofloxacin MBEC: 0.5 mg/mL) and *Bacillus* sp *Subtilis* Group 49 (Erythromycin: 0.125 mg/mL) (Table S4). The variance analysis indicated no biofilm age, source (CT), or isolation pattern (sessile or planktonic) effect in the susceptibility profile.

### The combination of antibiotics and enzymes was effective against biofilms

**Fig. 4:**
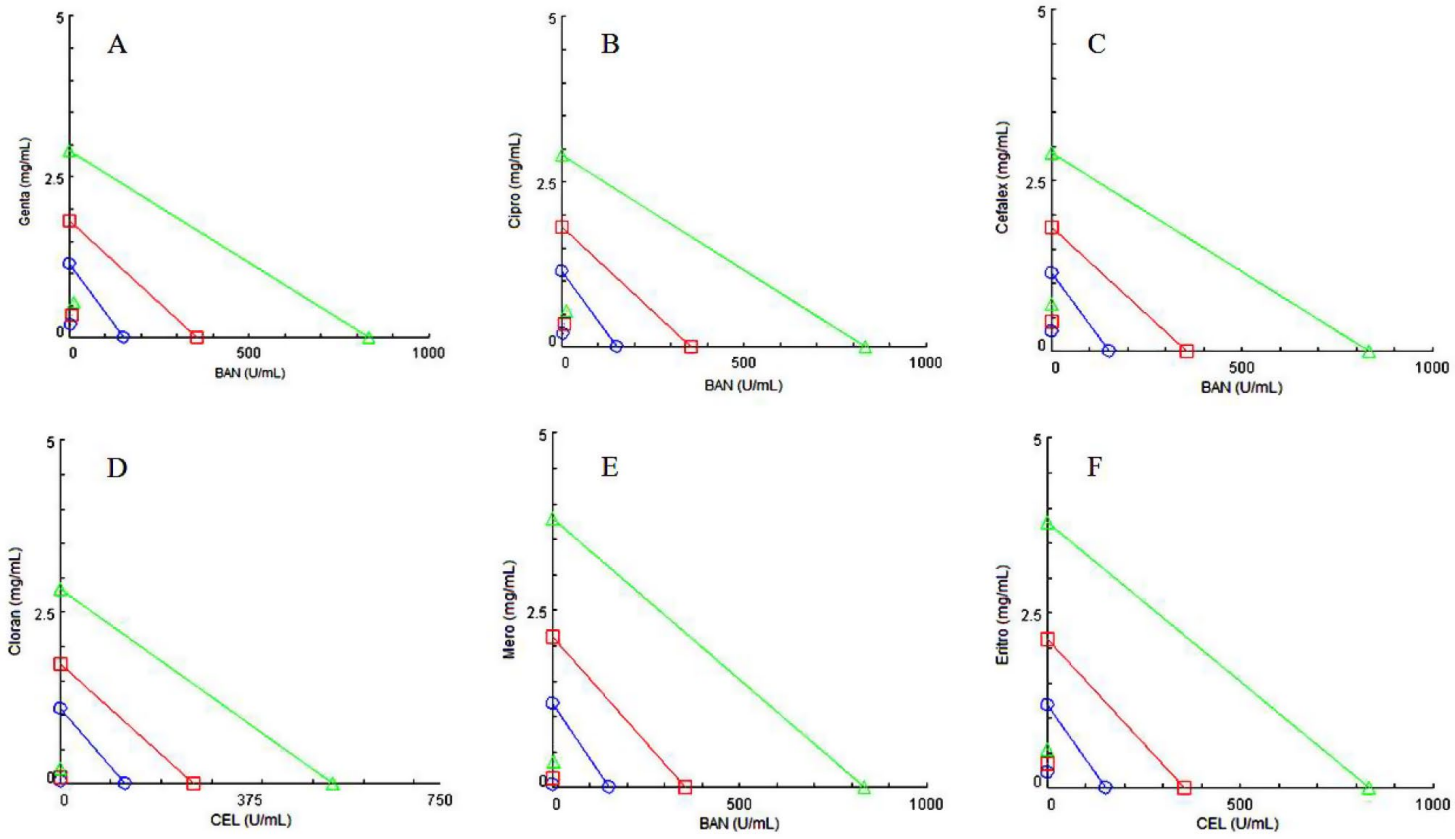
Isobolograms plotted with CompuSyn software. Lines indicate the combined concentrations with additive effect for 50 (blue markers, in circles), 75 (red markers, in squares) and 90% (green markers, in triangles) of the observed effect (biofilm eradication). Markers below the additive effect lines indicate synergistic effect of the combinations for the mentioned proportions. Points above the lines indicate antagonism of the combinations for the observed effect. Combinations: (**A**) Genta + BAN; (**B**) Cipro + BAN; (**C**) Cefalex + BAN; (**D**) Chloran + CEL; (**E**) Mero + BAN; (**F**) Erythro + CEL. Genta: Gentamicin; Cipro: Ciprofloxacin; Cefalex: Cephalexin; Chloran: Chloramphenicol; Mero: Meropenem; Erythro: Erythromycin; CEL: Cellulase

**Fig. 5:**
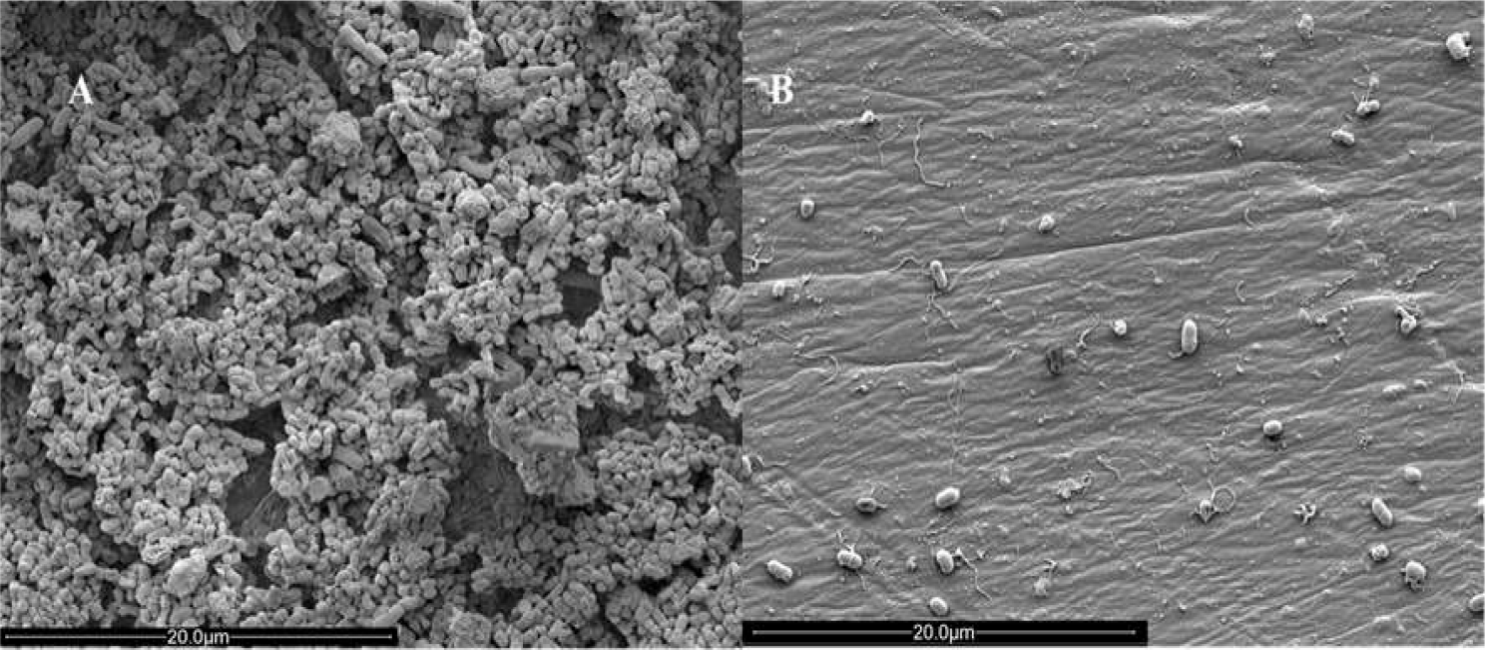
Effect of enzyme cellulase (200 U/mL, 4 h, 37 ºC) on the biofilm of *Stenotrophomonas maltophilia* 94. (**A**): untreated biofilm. (**B**): Biofilm treated with cellulase. Magnitude of photos: 7000x. Voltage: 2 kV

This strategy was used as a model to predict the combined use of enzymes and antibiotics against biofilms in industrial settings. We found six synergistic combinations (FIC < 0.5) out of the 10 tested (Table 2; Fig. 4). Three varieties showed no significant effect (FIC > 1), and the combination of AMG and meropenem showed an additive effect (0.5 < FIC < 1). Considering the more substantial impact of Cellulase in reducing biofilm biomass, biofilms of the bacterium *S. maltophilia* 94 treated and not treated with this enzyme were analyzed by scanning electron microscopy (Fig. 5).

**Table 2:** Effects of the combined use of enzyme and antimicrobial drugs over the isolates

Enzyme, antimicrobial drugs and isolate	MBEC				FIC		Result		
	Not combined		Combined						
AMG + Cef (*E. hormaechei* 125)		200 U/mL for enzymes and < 2 mg/mL for antimicrobial drugs		6,25 U/mL and 2 mg/mL		1,03		Indifferent	
AMG + Cef (*P. stutzeri* 110)				0,0156 U/mL and 1 mg/mL		1,01		Indifferent	
AMG + Chlor (*A. junii* 20)				6,25 U/mL and 2 mg/mL		1,03		Indifferent	
AMG + Gen (*E. meningoseptica* 8)				6,25 U/mL and 0,25 mg/mL		0.15		Synergism	
AMG + Mer (*K. cryocrescens* 40)				3,25 U/mL and 1 mg/mL		0.51		Additive	
BAN + Cef (*S. maltophilia* 94)				1,562 U/mL and 0,5 mg/mL		0.25		Synergism	
BAN + Cip (*E. mexicanum* 63)				0,78 U/mL and 0,125 mg/mL		0.06		Synergism	
BAN + Mer (*E. hormaechei* 152)				0,78 U/mL and 0,0625 mg/mL		0.03		Synergism	
Cellulase + Chlor (*L. sphaericus* 116)				0,78 U/mL and 0,125 mg/mL		0.06		Synergism	
Cellulase + Ery (*Bacillus* sp. Cereus group 1 157)				0,78 U/mL and 0,25 mg/mL		0.12		Synergism	

MBEC: Minimal biofilm eradication concentration. FIC: Fractionated inhibitory concentration. Gen: gentamicin; Ery: erythromycin; Cip: ciprofloxacin; Chlor: chloramphenicol; Cef: ceftriaxone. Mer: Meropenem; Cef: Ceftriaxone

### Biochemical characterization of microbial EPSs

The composition of the EPS extracted from the biofilm of the ten isolates evaluated by spectrophotometric methods consisted mainly of carbohydrates, showing that the extraction and purification method was good. A total of 15 sugar and amino sugars were identified by GC-MS (Table 3) on the EPS. The most frequent was glucose, which was present in all. Other carbohydrates were also found to be well represented in biofilm EPS. Mannose and erythrose were found in 6 of 8 strains, while arabinose and idose were found in 6 of 10. In contrast, some carbohydrates were detected in only one isolate, such as rhamnose in *Bacillus* sp. 157 (Cereus Group) and allose and those in *S. maltophilia* 94. Several types of target bonds for the action of the hydrolytic enzymes tested were detected in EPS (Table S4).

**Table 3:**
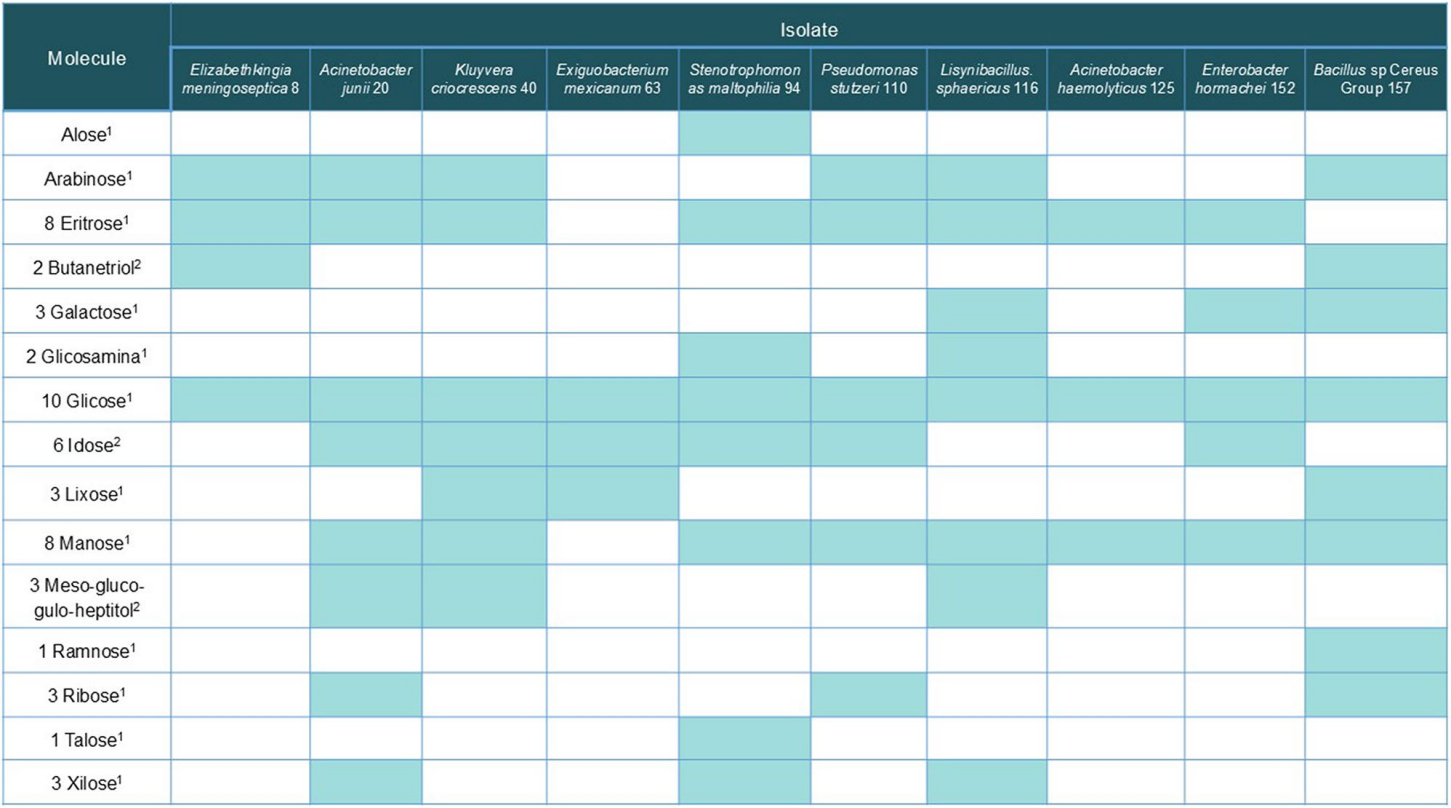
Glycosyl composition analysis of 10 selected isolates by GC-MS

Filled spaces in each column indicate detection of the molecule in question for each microorganism. Blank spaces indicate that there was no detection. 1: Carbohydrate; 2: synthesis intermediate

## Discussion

We have developed a novel strategy to address antibiotic resistance and biofilm formation by combining enzymes and antibiotics for a synergistic effect. Our research includes three critical experiments: examining the capability of enzymes to disrupt biofilms, evaluating the effectiveness of antibiotics against biofilms, and investigating their combined impact on dismantling biofilm structures. This comprehensive approach aims to enhance the efficacy of treatments against robust biofilm-related infections. This study is motivated by the urgent need to address the limitations of existing biofilm control strategies in CT, such as high toxicity and reduced effectiveness [14–16].

Combination therapies have grabbed the attention of worldwide researchers; several studies reported the antibiofilm activities of enzymes and antibiotics, even in vivo studies [38–43], but none of the studies evaluated this combined approach against CT-associated bacterial biofilms. Therefore, this is the first comprehensive study that uses enzymes and antibiotics alone or combined as antibiofilm strategies against the biofilms of bacteria isolated from CT water. The effectiveness of enzymatic biofilm treatment is reportedly influenced by various challenges, including tolerance to environmental conditions such as salinity, pH, and temperature extremes, as well as constraints related to the specificity and availability of substrates within the EPS [7]. To overcome these limitations, the enzymes we explored herein are genetically modified with higher stability. According to the data from the manufacturers, the polysaccharidases Ultrazym and AMG (exoenzymes) catalyze the cleavage of (1,4)-α-D-galacturonan methyl ester bonds and (1,4)- and (1,6)-α-D-glucose in non-reducing ends, respectively. The polysaccharidases BAN and cellulase (endoenzymes) hydrolyze (1,4)-α-D-glucose and 1,4-β-D-glucose bonds, respectively. DNase is an endonuclease that cleaves both single and double-stranded DNA, and Protease cleaves peptide bonds non-specifically.

Among tested enzymes, the polysaccharidases were highly active, which showed antibiofilm potential against 80% for all the isolates, and Cellulase presented the best results (*p* < 0.05) among all the tested enzymes. Variations in EPS composition help to explain interferences on substrate availability for the enzymes in the biofilms of the species investigated in this study [38]. Similar observations with the polysaccharidases BAN and AMG were reported. These enzymes were used to remove *Pseudomonas fluorescens* biofilms formed on polystyrene plates by adding glass fragments [39]. BAN and AMG reduced the biofilms’ biomass by 50% and 60%, respectively. Their increased efficacy can be explained by physical and chemical differences among the surfaces used in the adhesion assays [39].

In the assays of biochemical characterization of EPS of biofilms of 10 strongest biofilm producers’ bacteria, all carbohydrates indicated in Table 3 were detected in at least one bacteria isolated from water CTs except fructose and galactosamine (although the parent molecule, galactose, was detected). In some isolates, the detection of end products such as meso-gluco-gulo-heptitol, glucitol, and butanetriol is due to the partial or total derivatization of glucose with NaBH4. The carbohydrate profiles of the EPS of the isolates investigated in this study are similar to a few studies characterizing the EPS of environmental bacteria, especially from aquatic ecosystems, using chromatographic and spectrometric methods. For example, the presence of rhamnose, mannose, ribose, galactose, glucose, arabinose, fructose, xylose, and glucosamine was described in the EPS of *Bacillus* sp strains isolated from aquatic environments too [40, 41] Galactose, glucose, mannose, rhamnose, and arabinose were described in strains of species *A. Junni*, *A. baumannii*, or *A. calcoaceticus* [42, 43]. Poly-β-1,6-*N*-acetyl-glucosamine (PNAG) is one of the important building blocks in *A. baumannii*’s biofilm [44].

The presence of glucose and galactose on the EPS from *E. cloacae* was described by [45], while Iyer et al. [46], Hua et al. [45], and Paikra et al. [47] detected mannose, xylose, or fucose in other genera species. Many studies described the *S. maltophilia* EPS as a highly charged polymer due to the presence of three uronic acids and an ether-linked D-lactate substituent, showing 4-linked Glc, 4-linked GlcA and 3, 4-linked GalA in equal molar amounts in the native polymer [47] while Khan et al. [48] also detected uronic, acids (glucuronic acid (GlcA), galacturonic acid (GalA)) in *S. maltophilia* EPS alongside glucose. Linkage’s goal of the enzymes evaluated was detected in the bacterial isolates EPS (Table 3). The highly abundant EPS in biofilms protects bacteria from stress, environmental harshness, antimicrobials, and other undesirable agents [49].

In this study, the susceptibility of CT bacterial biofilms to different antibiotics was higher at 2 mg/mL. The complex biochemical composition of the EPS can outline a barrier to the diffusion of antimicrobials into biofilms, for instance, due to drug complexation with proteins or extracellular DNA (eDNA) [9]. Moreover, the stratified metabolic behavior of the sessile microorganisms can result in restrictions to oxygen flow and subsequent hypoxic regions in deeper layers of the biofilm, generating a spore-like state that impairs the action of drugs such as β-lactams and quinolones, which are more effective against bacteria with active metabolism [34, 50].

Antibiotics with even low MBEC could show potent anti-biofilm activities in combination with enzymes. Among ten varieties, only six were considered synergic and showed promising activity according to the mathematical criteria of Kumar et al. [47]. A probable explanation for the synergic interactions is the release of sessile microorganisms from the biofilms to the environment as the enzymes ruptured biofilms [51, 52]. Pirlar et al. reported that bacterial biofilms are degraded by several enzymes, such as trypsin, β-glucosidase, and DNase I [53]. In planktonic form, bacteria are more susceptible to antimicrobials in lower concentrations than those used in biofilm eradication [54]. It has been reported that biofilm eradication by enzymatic strategies allows antibiotics such as meropenem and amikacin to reduce microbial growth even in minimum doses [53]. In our studies, the combination of ciprofloxacin, BAN enzyme, and ofloxacin with proteases and serratiopeptidase has shown significant activity against biofilms of clinical isolates of *P. aeruginosa* and *S. epidermidis*. Biofilms of *S. epidermidis* become susceptible to ciprofloxacin only when treated with amylase and pectinase and to dicloxacillin and clindamycin when combined with pectinase [55]. The combination of cephalexin and BAN also showed synergistic effects against different strains. The cefamandole was also reported to have better efficacy with dispersin B against clinical isolates of *S. aureus* and *S. epidermidis* [56].

The lack of synergism in some combinations may be due to the low inhibitory concentrations of the antibiotics - promoting bacterial growth or the bacteria using the enzyme as a nutritional source [57]. Bacterial detachment from biofilms caused by enzymatic treatment can be followed by cell reattachment in the space of the well, given that biofilm growth is a dynamic cycle [38, 42]. Similarly, no significant differences were observed when comparing the combined use of pectinase and amoxicillin-clavulanate or ciprofloxacin against *S. aureus* and *P. aeruginosa* respectively compared the solo use of these antibiotics to the use of these drugs alone against these pathogens [38]. The drug-enzyme combination against *S. aureus* strains caused a 28% increase in the minimal inhibitory concentration (MIC) and minimal bactericidal concentration (MBC) values. Moreover, the drug-enzyme combination against *P. aeruginosa* strains increased the MIC and MBC by 89% and 92.8%, respectively. Interestingly, beyond the adverse effects on controlling bacterial growth, the enzyme increased bacterial adhesion compared to control samples [38].

The resistance of biofilms from bacterial strains isolated from aquatic settings like CT requires further examination. Our results align with existing studies indicating limited antimicrobial susceptibility in bacteria from various aquatic environments. Notably, biofilms from bacteria found in a drinking water treatment facility showed resistance to antibiotics such as colistin, meropenem, and piperacillin [58]. Poor biofilm susceptibility of enterobacteria isolated from potable water available at a hospital was observed to clindamycin, ampicillin, gentamicin, ciprofloxacin, erythromycin, and chloramphenicol [59]. In our study, the water treatment system may have contributed to this picture of poor antibiotic susceptibility of the isolates, as the retention time of water in physical filters may benefit horizontal gene transfer and multidrug (MDR) resistant bacteria [60]. It was described that a combined water treatment of ozonation and sand or activated carbon filtration weakly reduced the number of MDR *Staphylococcus* sp. strains, probably due to mechanisms associated with bacterial protection in deeper layers of the biofilms.

On the other hand, this system effectively decreased the number of MDR *Escherichia coli* and other *Enterobacteriaceae* strains [60]. The antibiotics utilized in this study are commonly employed in clinical practice, and their use in industrial systems may contribute to the development of antibiotic resistance. Nevertheless, this study is a valuable example of combining enzymes with antibiotics to enhance effectiveness. Future research will explore alternative antimicrobial agents, including other antibiotics, phages, polymers, and nanoparticles, to further expand and improve antimicrobial strategies.

## Conclusion

This work highlights research utilizing a microdilution technique inspired by the chequerboard method, revealing the synergistic effects of enzymes and various antibiotics, including aminoglycosides and β-lactams, against biofilms from pathogenic strains isolated from real-scale CTs. Given that synergy assessment methods for biofilms are yet to be fully standardized, we propose the methodology used in this study as a novel approach to evaluate the combined efficacy of antibiotics and enzymes. This strategy aims to control microbial biofilm growth, thereby mitigating the risks of infection, enhancing thermal exchange efficiency, and preventing structural complications in cooling systems. This integrated approach can effectively remove biofilms from other surfaces, including medical implants, hospital equipment, and materials.

## Electronic supplementary material

Below is the link to the electronic supplementary material.

Supplementary Material 1

## Data Availability

No datasets were generated or analysed during the current study.
