# Supplementary material for: The activity of hydrolytic enzymes and antibiotics against biofilms of bacteria isolated from industrial-scale cooling towers
Source: Microb Cell Fact. 2024 Oct 16;23:282. doi: 10.1186/s12934-024-02502-1 (PMC11484388; doi:10.1186/s12934-024-02502-1)
Supplement: Supplementary file 1 — Supplementary Material 1 [file 12934_2024_2502_MOESM1_ESM.docx]

Table S1. Biomass of biofilms treated with polysaccharidases, assessed through phenol-sulphuric method. Values are averages of at least three independent experiments and are expressed in μg/mL of total carbohydrates remaining after the treatment. The SD values are less than 13% of the media.

| **Bacterial origin** | | **Identity** | | **Control** | **AMG** | **Ultra** | **BAN** | **Celulase** |
| --- | --- | --- | --- | --- | --- | --- | --- | --- |
| **Source (CT)** | **Growth profile** |  |  | **(μg/mL)** | **(μg/mL)** | **(μg/mL)** | **(μg/mL)** | **(μg/mL)** |
| CT I | Planktonic (14 days) | *Bacillus sp.* Cereus group | 134 | 555.21 | 8.63 | 104.52 | 11.53 | 5.74 |
|  |  | *Bacillus sp.* Cereus group | 26 | 304.28 | 5.54 | 9.53 | 10.47 | 11.50 |
|  | Planktonic (21 days) | *Kluyvera cryocrescens* | 40 | 342.08 | 4.31 | 13.15 | 11.14 | 5.47 |
|  |  | *Lysinibacillus sphaericus* | 116 | 307.05 | 15.24 | 61.77 | 56.53 | 7.42 |
|  | Sessile (7 days) | *Acinetobacter radioresistens* | 104 | 331.09 | 7.51 | 140.93 | 8.10 | 58.79 |
|  |  | *Acinetobacter beijerinckii* | 107 | 524.17 | 2.73 | 7.76 | 6.83 | 8.43 |
|  |  | *Bacillus sp.* | 111 | 477.60 | 5.73 | 10.86 | 6.92 | 4.00 |
|  |  | *Bacillus sp.* Subtilis group | 131 | 542.03 | 16.98 | 87.37 | 7.87 | 3.08 |
|  |  | *Bacillus sp.* Cereus group | 138 | 310.41 | 9.47 | 2.52 | 4.11 | 11.11 |
|  |  | *Bacillus sp.* Cereus group | 23 | 323.84 | 2.87 | 8.02 | 8.35 | 7.78 |
|  | Sessile (14days) | *Bacillus sp.* Cereus group | 4 | 341.42 | 7.68 | 124.97 | 9.87 | 59.10 |
|  |  | *Bacillus sp.* Cereus group | 28 | 453.44 | 2.66 | 8.96 | 8.87 | 11.03 |
|  |  | *Acinetobacter junii* | 41 | 362.55 | 6.93 | 11.83 | 11.20 | 12.81 |
|  |  | *Bacillus sp.* Subtilis group | 55 | 514.09 | 6.51 | 9.89 | 5.01 | 1.86 |
|  |  | *Stenotrophomonas maltophilia* | 94 | 355.35 | 25.55 | 76.27 | 6.23 | 155.83 |
|  |  | *Bacillus sp* Cereus group | 95 | 510.15 | 11.23 | 100.65 | 13.85 | 3.19 |
|  |  | *Acinetobacter junii* | 97 | 448.50 | 7.72 | 0.30 | 4.25 | 3.19 |
|  | Sessile (21 days) | *Bacillus sp.* Subtilis group | 17 | 316.42 | 5.85 | 125.77 | 8.63 | 116.92 |
|  |  | *Bacillus sp.* | 43 | 460.79 | 13.16 | 131.11 | 5.92 | 41.45 |
| CT II | Planktonic (7 days) | *Bacillus sp.* | 30 | 304.27 | 5.13 | 10.94 | 11.05 | 12.44 |
|  |  | *Bacillus sp* Cereus group | 66 | 550.47 | 6.23 | 94.24 | 14.20 | 6.87 |
|  |  | *Staphylococcus epidermidis* | 72 | 477.01 | 9.47 | 2.52 | 4.11 | 11.11 |
|  |  | *Enterobacter hormaechei* | 108 | 574.91 | 5.24 | 8.31 | 6.74 | 1.86 |
|  |  | *Acinetobacter haemolyticus* | 125 | 531.51 | 4.28 | 9.17 | 6.34 | 6.12 |
|  | Planktonic (14days) | *Elizabethkingia meningoseptica* | 8 | 351.17 | 5.63 | 9.71 | 12.00 | 16.10 |
|  |  | *Acinetobacter haemolyticus* | 9 | 243.71 | 5.81 | 121.60 | 9.27 | 121.83 |
|  |  | *Bacillus sp* Subtilis group | 10 | 520.61 | 10.75 | 116.61 | 7.91 | 118.96 |
|  |  | *Acinetobacter junii* | 20 | 494.03 | 6.31 | 114.37 | 70.18 | 164.64 |
|  |  | *Bacillus sp.* Cereus group | 44 | 557.61 | 7.34 | 96.59 | 135.01 | 6.53 |
|  |  | *Geobacillus stearothermophilus* | 45 | 283.00 | 6.20 | 102.25 | 8.29 | 142.13 |
|  |  | *Bacillus sp* Subtilis group | 49 | 345.03 | 4.68 | 9.05 | 5.66 | 5.62 |
|  |  | *Enterobacter hormaechei* | 152 | 437.57 | 5.34 | 136.15 | 5.49 | 10.51 |
|  | Planktonic (21 days) | *Bacillus sp.* Cereus group | 157 | 349.52 | 8.27 | 92.73 | 8.29 | 7.66 |
|  | Sessile (7 days) | *Bacillus sp.* | 25 | 267.41 | 4.88 | 10.36 | 5.36 | 0.52 |
|  |  | *Bacillus sp.* Cereus group | 38 | 289.63 | 5.62 | 11.19 | 11.24 | 10.63 |
|  |  | *Exiguobacterium mexicanum* | 63 | 518.96 | 94.32 | 128.97 | 8.63 | 106.54 |
|  |  | *Bacillus sp* Cereus group | 86 | 282.04 | 14.41 | 175.10 | 4.02 | 9.91 |
|  |  | *Bacillus circulans* | 102 | 494.68 | 6.51 | 9.89 | 5.01 | 1.86 |
|  |  | *Bacillus sp.* | 106 | 287.33 | 6.23 | 94.24 | 14.20 | 6.87 |
|  |  | *Pseudomonas stutzeri* | 110 | 248.14 | 1.72 | 9.24 | 4.85 | 10.32 |
|  | Sessile (14 days) | *Bacillus sp.* | 1 | 474.08 | 8.14 | 102.38 | 8.68 | 3.50 |
|  |  | *Bacillus sp* Cereus group | 11 | 248.18 | 4.34 | 130.09 | 9.23 | 54.38 |
|  |  | *Acinetobacter junii* | 34 | 520.36 | 3.01 | 10.22 | 8.23 | 11.40 |
|  |  | *Bacillus sp.* | 83 | 468.06 | 11.20 | 98.93 | 7.22 | 84.49 |
|  |  | *Bacillus sp.* | 112 | 326.53 | 2.56 | 16.64 | 2.98 | 16.80 |
|  |  | *Acinetobacter haemolyticus* | 118 | 352.06 | 8.18 | 129.31 | 8.34 | 7.22 |
|  |  | *Bacillus sp.* Subtilis group | 140 | 364.94 | 6.23 | 94.24 | 14.20 | 6.87 |
|  |  | *Bacillus sp.* | 143 | 332.13 | 6.08 | 132.12 | 9.08 | 9.48 |
|  |  | *Bacillus sp.* Subtilis group | 147 | 489.07 | 2.73 | 7.76 | 6.83 | 8.43 |
|  | Sessile (21 days) | *Bacillus sp.* | 91 | 306.54 | 12.08 | 55.21 | 6.49 | 5.29 |

Table S2. Biomass of biofilms treated with DNAse and Protease (crystal violet staining). Values are averages of at least three independent experiments and are expressed as absorbance values (590 nm) with and without treatment. The SD values are less than 12% of the media.

| **Bacterial origin** | | **Identity** | | **Control** | **DNAse** | **Protease** |
| --- | --- | --- | --- | --- | --- | --- |
| **Source (CT)** | **Growth profile** |  |  | (Abs) | (Abs) | (Abs) |
| CT I | Planktonic (14 days) | *Bacillus sp.* | 26 | 0.850 | 0.294 | 0.362 |
|  | Planktonic (21 days) | *Bacillus sp.* Cereus group | 40 | 0.974 | 0.535 | 0.363 |
|  |  | *Bacillus sp.* | 116 | 0.675 | 0.370 | 0.476 |
|  | Sessile (7 days) | *Acinetobacter junii* | 104 | 0.919 | 0.731 | 0.408 |
|  |  | *Acinetobacter radioresistens* | 107 | 0.807 | 0.627 | 0.609 |
|  |  | *Pseudomonas stutzeri* | 111 | 0.691 | 0.688 | 0.665 |
|  |  | *Acinetobacter haemolyticus* | 131 | 0.717 | 0.340 | 0.398 |
|  |  | *Bacillus sp.* Cereus group | 138 | 0.913 | 0.890 | 0.439 |
|  |  | *Brevibacterium halotolerans* | 23 | 0.657 | 0.299 | 0.282 |
|  | Sessile (14days) | *Bacillus sp.* Subtilis group | 55 | 0.948 | 0.544 | 0.364 |
|  |  | *Bacillus sp.* Cereus group | 94 | 0.813 | 0.424 | 0.386 |
|  |  | *Stenotrophomonas maltophilia* | 97 | 0.711 | 0.468 | 0.332 |
|  |  | *Bacillus sp.* Cereus group | 4 | 0.827 | 0.433 | 0.363 |
|  |  | *Bacillus sp.* Cereus group | 28 | 0.821 | 0.378 | 0.477 |
|  |  | *Kluyvera cryocrescens* | 41 | 0.977 | 0.330 | 0.368 |
|  |  | *Bacillus sp.* | 95 | 0.760 | 0.578 | 0.409 |
|  | Sessile (21 days) | *Acinetobacter junii* | 43 | 0.958 | 0.712 | 0.625 |
|  |  | *Bacillus sp.* Subtilis group | 17 | 0.596 | 0.243 | 0.308 |
| CT II | Planktonic (7 days) | *Exiguobacterium mexicanum* | 66 | 0.988 | 0.302 | 0.141 |
|  |  | *Bacillus sp.* Cereus group | 72 | 0.679 | 0.394 | 0.295 |
|  |  | *Acinetobacter beijerinckii* | 108 | 0.819 | 0.802 | 0.568 |
|  |  | *Bacillus sp.* Subtilis group | 135 | 0.722 | 0.411 | 0.460 |
|  |  | *Bacillus sp.* Cereus group | 30 | 0.973 | 0.389 | 0.323 |
|  |  | *Acinetobacter haemolyticus* | 125 | 0.728 | 0.436 | 0.527 |
|  | Planktonic (14days) | *Elizabethkingia meningoseptica* | 8 | 0.784 | 0.324 | 0.442 |
|  |  | *Acinetobacter haemolyticus* | 9 | 0.786 | 0.241 | 0.407 |
|  |  | *Bacillus sp* Subtilis group | 10 | 0.779 | 0.344 | 0.359 |
|  |  | *Acinetobacter junii* | 20 | 0.756 | 0.357 | 0.356 |
|  |  | *Bacillus sp.* Cereus group | 45 | 0.963 | 0.272 | 0.190 |
|  |  | *Geobacillus stearothermophilus* | 49 | 0.957 | 0.491 | 0.300 |
|  |  | *Enterobacter hormaechei* | 152 | 0.679 | 0.291 | 0.296 |
|  |  | *Bacillus sp.* | 44 | 0.929 | 0.429 | 0.385 |
|  | Planktonic (21 days) | *Bacillus sp.* Cereus group | 157 | 0.642 | 0.453 | 0.453 |
|  | Sessile (7 days) | *Bacillus sp.* Cereus group | 25 | 0.744 | 0.257 | 0.270 |
|  |  | *Bacillus sp.* Subtilis group | 63 | 0.990 | 0.462 | 0.236 |
|  |  | *Bacillus sp.* Cereus group | 102 | 0.654 | 0.532 | 0.430 |
|  |  | *Bacillus circulans* | 106 | 0.648 | 0.535 | 0.508 |
|  |  | *Enterobacter hormaechei* | 110 | 0.820 | 0.711 | 0.387 |
|  |  | *Bacillus sp.* Cereus group | 38 | 0.977 | 0.315 | 0.414 |
|  |  | *Acinetobacter junii* | 86 | 0.794 | 0.456 | 0.307 |
|  | Sessile (14 days) | *Bacillus sp.* | 34 | 0.985 | 0.467 | 0.492 |
|  |  | *Staphylococcus epidermidis* | 82 | 0.777 | 0.536 | 0.417 |
|  |  | *Bacillus sp.* | 112 | 0.598 | 0.514 | 0.540 |
|  |  | *Bacillus sp.* | 1 | 0.850 | 0.381 | 0.423 |
|  |  | *Bacillus sp* Cereus group | 11 | 0.635 | 0.358 | 0.387 |
|  |  | *Bacillus sp.* Subtilis group | 140 | 0.525 | 0.295 | 0.472 |
|  |  | *Bacillus sp.* | 143 | 0.770 | 0.224 | 0.206 |
|  |  | *Bacillus sp.* Subtilis group | 147 | 0.819 | 0.473 | 0.403 |
|  |  | *Lysinibacillus sphaericus* | 118 | 0.853 | 0.828 | 0.374 |
|  | Sessile (21 days) | *Bacillus sp.* | 91 | 0.618 | 0.405 | 0.349 |

Table S3. Percentage of isolates with MBEC >2 mg/mL and MBEC <2 mg/mL with respect to biofilm age, CT and sessile and planktonic profile.

| **Biofilm age (days)** | **Gentamicin** | | **Erythromycin** | | **Ciprofloxacin** | | **Chloramphenicol** | | **Ceftriaxone** | |
| --- | --- | --- | --- | --- | --- | --- | --- | --- | --- | --- |
|  | MBEC > 2 | MBEC < 2 | MBEC > 2 | MBEC < 2 | MBEC < 2 | MBEC < 2 | MBEC > 2 | MBEC< 2 | MBEC > 2 | MBEC < 2 |
| 7 | 73% | 27% | 77% | 23% | 41% | 59% | 54.5% | 45.5% | 73% | 37% |
| 14 | 77% | 23% | 68% | 32% | 59% | 41% | 59% | 41% | 82% | 18% |
| 21 | 83% | 17% | 50% | 50% | 83% | 17% | 33.3% | 66.7% | 67% | 33% |
|  |  |  |  |  |  |  |  |  |  |  |
| **CT type** | **Gentamicin** | | **Erythromycin** | | **Ciprofloxacin** | | **Chloramphenicol** | | **Ceftriaxone** | |
|  | MBEC > 2 | MBEC < 2 | MBEC > 2 | MBEC < 2 | MBEC > 2 | MBEC < 2 | MBEC > 2 | MBEC < 2 | MBEC >2 | MBEC < 2 |
|  |  |  |  |  |  |  |  |  |  |  |
| CTI | 78% | 22% | 55.5% | 44.5% | 67% | 33% | 61% | 39% | 78% | 22% |
| CTII | 75% | 25% | 78% | 22% | 47% | 53% | 53% | 47% | 75% | 25% |
|  |  |  |  |  |  |  |  |  |  |  |
| **Growth profile** | **Gentamicin** | | **Erythromycin** | | **Ciprofloxacin** | | **Chloramphenicol** | | **Ceftriaxone** | |
|  | MBEC >2 | MBEC < 2 | MBEC > 2 | MBEC < 2 | MBEC > 2 | MBEC < 2 | MBEC >2 | MBEC < 2 | MBEC >2 | MBEC < 2 |
| Sessile | 73% | 27% | 64% | 36% | 51.5% | 48.5% | 57.6% | 42.4% | 70% | 30% |
| Planktonic | 82% | 18% | 82% | 18% | 59% | 41% | 47% | 53% | 88% | 12% |

All isolates showing MBEC > 2 mg/mL for meropenem and cephalexin.

Table S4. Minimal biofilm eradication concentration (MBEC) of antibiotics against 50 bacterial isolates.

| **Bacterial origin** | | **Identity** | | **Antibiotic** | | | | | |
| --- | --- | --- | --- | --- | --- | --- | --- | --- | --- |
| **Source (CT)** | **Growth profile** |  |  | **Gen** | **Ery** | **Cip** | **Chlor** | **Cef** |  |
| CT I | Planktonic (14 days) | *Bacillus sp.* Cereus group | 26 | < 2 | 1 | < 2 | < 2 | 1 |  |
|  | Planktonic (21 days) | *Kluyvera cryocrescens* | 40 | 1 | < 2 | < 2 | 2 | < 2 |  |
|  |  | *Lysinibacillus sphaericus* | 116 | < 2 | < 1 | < 2 | < 2 | < 2 |  |
|  | Sessile (7 days) | *Bacillus sp.* Cereus group | 23 | 2 | < 2 | 1 | < 2 | < 2 |  |
|  |  | *Acinetobacter radioresistens* | 104 | < 2 | < 2 | 1 | < 2 | < 2 |  |
|  |  | *Acinetobacter beijerinckii* | 107 | < 2 | < 2 | 2 | < 2 | < 2 |  |
|  |  | *Bacillus sp.* Cereus group | 138 | < 2 | 1 | < 2 | < 2 | 1 |  |
|  | Sessile (14days) | *Bacillus sp.* Cereus group | 4 | < 2 | < 2 | < 2 | 2 | < 2 |  |
|  |  | *Bacillus sp.* Cereus group | 28 | 1 | < 2 | < 2 | < 2 | 1 |  |
|  |  | *Acinetobacter junii* | 41 | < 2 | 2 | < 2 | < 2 | < 2 |  |
|  |  | *Bacillus sp.* Subtilis group | 55 | 1 | < 2 | < 2 | < 2 | < 2 |  |
|  |  | *Stenotrophomonas maltophilia* | 94 | < 2 | 2 | 1 | < 2 | < 2 |  |
|  |  | *Bacillus sp* Cereus group | 95 | 2 | 1 | < 2 | < 2 | < 2 |  |
|  |  | *Acinetobacter junii* | 97 | 2 | < 2 | < 2 | < 2 | 1 |  |
|  |  | *Bacillus sp.* | 111 | < 2 | < 2 | < 2 | 2 | < 2 |  |
|  | Sessile (21 days) | *Bacillus sp.* Subtilis group | 17 | < 2 | 1 | < 2 | 1 | < 2 |  |
|  |  | *Bacillus sp.* | 43 | < 2 | < 2 | < 2 | 2 | 1 |  |
| CT II | Planktonic (7 days) | *Bacillus sp.* | 30 | < 2 | < 2 | 1 | 1 | < 2 |  |
|  |  | *Bacillus sp* Cereus group | 66 | < 2 | < 2 | < 2 | 2 | < 2 |  |
|  |  | *Staphylococcus epidermidis* | 72 | < 2 | < 2 | 1 | < 2 | 1 |  |
|  |  | *Enterobacter hormaechei* | 108 | 2 | 2 | 2 | 2 | 2 |  |
|  |  | *Acinetobacter haemolyticus* | 125 | < 2 | < 2 | < 2 | 1 | 1 |  |
|  | Planktonic (14 days) | *Bacillus sp* Subtilis group | 49 | < 2 | > 1 | < 2 | < 2 | < 2 |  |
|  |  | *Elizabethkingia meningoseptica* | 8 | < 2 | 1 | < 2 | < 2 | < 2 |  |
|  |  | *Acinetobacter haemolyticus* | 9 | < 2 | < 2 | 2 | < 2 | < 2 |  |
|  |  | *Bacillus sp* Subtilis group | 10 | < 2 | < 2 | < 2 | 1 | < 2 |  |
|  |  | *Acinetobacter junii* | 20 | 1 | < 2 | < 2 | 1 | < 2 |  |
|  |  | *Brevibacterium halotolerans* | 21 | < 2 | 1 | 1 | < 2 | 1 |  |
|  |  | *Bacillus sp.* Cereus group | 44 | < 2 | < 2 | 2 | < 2 | < 2 |  |
|  |  | *Geobacillus stearothermophilus* | 45 | < 2 | < 2 | 1 | 1 | < 2 |  |
|  |  | *Enterobacter hormaechei* | 152 | < 2 | < 2 | 1 | 2 | < 2 |  |
|  | Planktonic (21 days) | *Bacillus sp.* Cereus group | 157 | < 2 | < 2 | 2 | < 2 | 1 |  |
|  | Sessile (7 days) | *Bacillus sp.* | 25 | 2 | < 2 | < 2 | < 2 | < 2 |  |
|  |  | *Bacillus sp.* Cereus group | 38 | < 2 | < 2 | 2 | 1 | < 2 |  |
|  |  | *Exiguobacterium mexicanum* | 63 | < 2 | < 2 | 1 | 2 | < 2 |  |
|  |  | *Bacillus sp* Cereus group | 86 | < 2 | < 2 | 1 | 1 | < 2 |  |
|  |  | *Bacillus circulans* | 102 | < 2 | < 2 | 1 | 1 | < 2 |  |
|  |  | *Pseudomonas stutzeri* | 110 | < 2 | < 2 | < 1 | < 2 | < 2 |  |
|  | Sessile (14 days) | *Bacillus sp.* | 1 | < 2 | < 2 | < 2 | 2 | < 2 |  |
|  |  | *Bacillus sp* Cereus group | 11 | < 2 | < 2 | 2 | 1 | < 2 |  |
|  |  | *Acinetobacter junii* | 34 | 2 | < 2 | 1 | < 2 | < 2 |  |
|  |  | *Staphylococcus equorum* | 82 | 1 | < 2 | < 2 | 1 | 1 |  |
|  |  | *Bacillus sp.* | 83 | < 2 | < 2 | 1 | < 2 | 1 |  |
|  |  | *Bacillus sp.* | 112 | < 2 | 1 | < 2 | < 2 | < 2 |  |
|  |  | *Acinetobacter haemolyticus* | 118 | < 2 | 1 | < 2 | < 2 | < 2 |  |
|  |  | *Bacillus sp.* Subtilis group | 140 | < 2 | 1 | 2 | < 2 | < 2 |  |
|  |  | *Bacillus sp.* | 143 | < 2 | < 2 | 1 | < 2 | < 2 |  |
|  |  | *Bacillus sp.* Subtilis group | 147 | < 2 | < 2 | < 2 | 1 | < 2 |  |
|  | Sessile (21 days) | *Bacillus sp.* | 91 | < 2 | 2 | < 2 | 2 | < 2 |  |

Legend: Gen: gentamicin; Ery: erythromycin; Cip: ciprofloxacin; Chlor: chloramphenicol; Cef: ceftriaxone. All isolates presented MBEC > 2 mg/mL for meropenem and for cephalexin, and thus, are not shown in this table.

Table S5. Fragmentation profiles and glycosidic bonds identified in PMAA by GC-MS.

| **Bacterial isolates** | **Fragments or terminal links ^1^** | **Retention time**^2^ |
| --- | --- | --- |
| *Elizabethkingia meningoseptica* 8 | 115, 145, 187, 217, 289 | 25715, 25875, 29005 |
|  | t-ara | 23755 |
|  | 4-man | 26000 |
| *Acinetobacter junii* 20 | 115, 145, 147, 187, 289, 321, 325 | 21940, 24125, 24610, 28120, 29250, 29390, 29345 |
|  | t-ara | 21500, 2400 |
|  | t-glic | 25905, 27705, 28005 |
|  | t-xil | 18385 |
|  | 4-man | 25725, 26005 |
| *Kluyvera cryocrescens* 40 | 115, 145, 187, 289 | 24120, 24600, 29245, 29335 |
| *Exiguobacterium mexicanum* 63 | 115, 145, 187, 217, 289 | 24125 |
|  | 4-lix | 23775 |
|  | t-glic | 27705, 27985 |
| *Stenotrophomonas maltophilia* 94 | 115, 128, 218, 289, 326, 421 | 21955, 24610 |
|  | 4-lix | 23775 |
|  | t-glic | 27705, 27985 |
| *Pseudomonas stutzeri* 110 | 115, 145, 187, 217, 289, 425 | 29135, 29250 |
|  | t-ara | 21950 |
|  | t-glic | 27705, 27985 |
| *Lysinibacillus sphaericus* 116 | 115, 128, 145, 187, 217, 289 | 21375, 23900, 24145 |
|  | t-ara | 21510 |
|  | t-xil | 23550 |
|  | 4-man | 25720, 26015 |
|  | t-man | 25730, 25960 |
| *Acinetobacter haemolyticus* 125 | 115, 145, 189, 217, 289, 420 | 24130, 24610, 29135 |
|  | 4-man | 25720, 26015 |
|  | t-glic | 27705, 27985 |
| *Enterobacter hormaechei* 152 | 115, 145, 187, 217, 289, 321, 424 | 24125, 24610, 24620, 29255 |
|  | t-glic | 27705, 27985 |
| *Bacillus* sp Cereus Group 157 | 115, 145, 187, 217, 289, 422 | 29125, 29240 |
|  | t-glic | 27965 |

^1^: m/z values. ^2^: 30 m PTE-5 column, samples prepared in dichloromethane. Fragments of m/z = 115 and 145 are suggestive of the presence of 1-2 and 1-4 bonds. Fragments of m/z = 145, 187 and 289 are suggestive of the presence of 1-3 bonds. The fragment of m/z = 422 indicates bonds 1-6.
